# Preoperative Smoking Cessation in Chronic Respiratory Disease: A Community-Based Approach Guided by the Transtheoretical Model

**DOI:** 10.7759/cureus.97499

**Published:** 2025-11-22

**Authors:** Ata Mohajer-Bastami, Sarah Moin, Lucy Tweedlie, Ahmed Ahmed, Anurag Agarwal, Anil Lala, Edgar Gelber, Graham Whiteley, Sjaak Pouwels, Ameer Khamise, Ali W Khalid, Aya Khalid, Wah Yang, Bijendra Patel, Suhaib Ahmad

**Affiliations:** 1 Department of Surgery, Ealing Hospital, London, GBR; 2 Department of Surgery, East Surrey Hospital, London, GBR; 3 Department of Psychology, Imperial College London, London, GBR; 4 Department of General Surgery, Imperial College London, London, GBR; 5 Department of Surgery, Betsi Cadwaladr University Health Board, Bangor, GBR; 6 Department of Intensive Care Medicine, Elisabeth-Tweesteden Hospital, Tilburg, NLD; 7 School of Medicine, The University of Buckingham, Buckingham, GBR; 8 School of Medicine, Medipol University International School of Medicine, Istanbul, TUR; 9 Department of Surgery, The First Affiliated Hospital of Jinan University, Guangzhou, CHN; 10 Department of Surgical Science, Barts Cancer Institute, London, GBR; 11 Department of Surgery, Health Education and Improvement Wales (HEIW), Swansea, GBR

**Keywords:** behaviour change, chronic respiratory disease, preoperative care, smoking cessation, transtheoretical model

## Abstract

This study critically evaluates the Transtheoretical Model (TTM) of behaviour change and proposes a tailored smoking cessation intervention for adults with chronic respiratory disease, a high-risk group for poor surgical outcomes. Through a narrative review and critical appraisal of TTM, a targeted intervention was developed, integrating behavioural theory and motivational interviewing within community-based healthcare and preoperative care settings. The proposed intervention, delivered through local “support hubs,” is stage-specific and designed to enhance readiness for change by combining motivational interviewing, self-efficacy reinforcement, and decisional balance strategies. Theoretical modelling was informed by behavioural literature and case examples such as the Salford Hub, ensuring contextual relevance and feasibility. The intervention is anticipated to improve engagement and quit rates while enhancing accessibility and reducing disparities in care for individuals with conditions such as chronic obstructive pulmonary disease (COPD) and asthma. Although long-term outcomes require further study, existing evidence supports the short-term benefits of such behavioural approaches. Overall, the TTM provides a flexible and personalised framework for smoking cessation that, when combined with motivational interviewing and community-based delivery, may significantly improve outcomes for high-risk surgical patients with chronic respiratory disease. Although long-term outcomes require further study, the proposed framework may improve short-term engagement and quit rates; further implementation research is warranted.

## Introduction and background

The Transtheoretical Model (TTM): foundations and clinical relevance

Cigarette smoking accounts for the vast majority (around 90%) of chronic obstructive pulmonary disease (COPD) cases [1]. Several studies have shown that cigarette smoke has an adverse effect on established lung conditions, including asthma and COPD, in their response to therapy [2].

The Transtheoretical Model (TTM), also referred to as the Stages of Change Theory, was initially developed as a stage-step theoretical framework that includes “stages” an individual can move forwards and backwards through during a change in health behaviour [3]. As with any model, it can be applied practically when attempting to more strongly consider a relevant change. The model also aims to identify barriers to inform public health policy. More recent research suggested a cyclical adaptation of the model is a more accurate representation, demonstrating that individuals often cycle through change stages multiple times rather than progressing linearly, as many require repeated attempts before sustained change [4]. The theory aims to understand the influences of people’s behaviours and describes how individuals can move dynamically through the different stages of change. Table 1 shows the different stages of the model [3]. Each stage represents the readiness of the individual to commit to changing a particular health behaviour.

**Table 1:** Stages of Human Behaviour Change

Stage	Description	Key processes/focus
Pre-contemplation	Unaware or not ready to change; may deny the problem	Increase awareness through education and feedback
Contemplation	Aware of the problem and considering change, but undecided	Explore pros and cons; encourage self-reflection
Preparation	Intends to change soon and may take small initial steps	Strengthen commitment; set goals and plan actions
Action	Actively modifying behaviour and environment	Apply coping strategies, reinforcement, and support
Maintenance	Sustaining new behaviour and preventing relapse	Strengthen habits, monitor progress, and maintain support
Relapse	Reverting to old behaviour after progress	Identify triggers; renew motivation and restart the cycle

Maintenance is the continued and maintained abstinence from the changed behaviour and the ability to avoid temptations to relapse back into the behaviour. Relapse can occur at any stage of the model [5]. According to the model, individuals do not skip any stages but rather progress or regress through each stage sequentially. The following section provides a critical analysis of the Transtheoretical Model in general terms before considering its application to a smoking cessation intervention.

The Transtheoretical Model revisited: evidence and limitations

Model Overview

TTM was originally applied to addictive behaviours, including smoking [5]. Since then, it has continued to remain a widely used cognitive-behavioural approach in targeting smoking cessation. Changing health-related behaviour is complex; biopsychosocial determinants (social and psychological factors) shape readiness and response and should inform tailoring [6-8]. It is therefore important to consider health conditions from a biopsychosocial perspective before tailoring an intervention [9].

Benefits of the Model

One review considered the usefulness of the model when looking into promoting physical activity. The paper found that at each stage of change, individuals use different strategies and techniques [9]. That is to say that the intervention would differ from a person who has not considered quitting smoking, compared to someone ready to quit but has not yet attempted. Although this can be seen as a limitation as it will be more difficult to assess which intervention at which stage is the most effective, the very dynamic and flexible nature of this model reflects the complexity of changes of emotions and feelings individuals experience at each stage of this model [8]; hence, one can attempt to tailor the intervention at each stage to add encouragement and produce better results [10].

Furthermore, as with other behavioural theories, the very nature of the behavioural theory provides a framework to be utilised in helping to design an intervention [9]. One can also use the same framework in evaluating and adapting interventions as required to suit the context of the intervention. Further to aiding in designing interventions, one can use the framework to predict intervention outcomes. This was observed in a review of using the Transtheoretical Model to treat eating disorders, which found sufficient evidence to suggest that, for various variables, initial change is predictive of intervention outcomes [11,12], which can help evaluate the usefulness of each intervention applied. Smoking itself has a wide range of variable factors and barriers that prevent change, thus having a framework that can be applied to both plan and measure effective intervention outcomes to stop smoking. 

Limitations of the Model

Despite its benefits, there are marked limitations to applying the Transtheoretical Model to changing behaviour and proposing interventions. The model represents a relative “one-size-fits-all” framework. Its robust nature carries with it the assumption that, at each stage, an individual will be going through the same or similar experiences. That would result in the assumption that the barriers for each individual are the same. This negates the impact of wider determinants of health, such as social and environmental factors, which will not be the same for everyone. Social determinants of health and behaviours are important and have not been considered within the framework [10].

Other criticisms of the model are due to the implicit assumption that all individuals behave rationally. It is assumed and implied that individuals make reasonable, methodical follow-up plans, which is not always the case [13]. It neglects how people may sometimes behave irrationally and does not consider deep-rooted habits, which perhaps can be more unshakable. Furthermore, the model appears to lack validity in how to tackle more complex health-related behaviours. In a Finnish study conducted to utilise the model to assess the readiness of patients with recently diagnosed diabetes for dietary change at the beginning of counselling, it was found that determining stages of change through examining counselling can be difficult [14]. Without clarity of stage assessment, it would be more difficult to tailor an intervention to target the correct stage of change.

Evidence Base

A more widespread criticism of behavioural theories, including the Transtheoretical Model, is that there is a weak evidence base for their success in changing behaviours and generating successful interventions with their use. Particularly with the Transtheoretical Model, there has been minimal supportive evidence to justify its ongoing use [15]. Adams and White, experts in public health, argue the same [14]. They go on to mention that previous interventions based on the Transtheoretical Model have likely failed to appreciate the complexity of the task of changing a behaviour.

Community Application

Several arguments have been made that stage-based targeted intervention will more likely bring on short-term effects. Multiple studies confirm this, such as the most recent studies and systematic reviews looking at using the model for smoking cessation interventions, as well as more recent systematic reviews that did suggest positive outcomes utilising the model, particularly amongst adolescents and individuals in middle- or low-income countries [16-18]. They found that the effects for short-term outcomes were good, but not so for long-term effectiveness. From a public health perspective, for a health behaviour change to have a positive public health impact, the effects should ideally be long-lasting rather than having short-term positive outcomes [19]. Thus, if any intervention based on the model will bring only short-term effects most of the time, there may be a lack of an overall long-term purpose for its use.

Self-efficacy is an individual’s belief that they can carry out and maintain a new behaviour. Interventions based on the Transtheoretical Model can be seen to be more successful relative to the level of one’s self-efficacy. Therefore, messages based on data collected on self-efficacy information can be effectively used. Previous research has suggested that promoting self-efficacy moved people from contemplation to preparation but did not move people from pre-contemplation to contemplation [20,21]. This can be potentially limiting in knowing which effective intervention to implement at which step. Conversely, more recent studies have suggested that self-efficacy reinforcement can be effective at all stages. Therefore, there may be an important role for self-efficacy information gathered to promote positive persuasive messages at all stages of the model [5]. This paper will now provide an overview of a public health smoking cessation intervention for the target group described based on the Transtheoretical Model and provide a critical analysis of the suggested intervention.

Methodology

A narrative review was conducted using Ovid MEDLINE, Scopus, and Google Scholar to identify relevant literature on the use of the Transtheoretical Model and smoking cessation. The following keywords and MeSH terms were used in combinations: “transtheoretical model,” “stages of change,” “chronic respiratory disease,” “asthma,” “COPD,” “preoperative,” “high-risk,” “self-efficacy,” “prevention,” and “community health service.”

Boolean operators (AND and OR) were applied to refine the results.

Inclusion Criteria

Inclusion criteria included English-language articles; studies discussing clinical applications, mechanisms, or outcomes of the model on patients reducing or stopping smoking; and case reports, narrative, or systematic reviews.

Exclusion Criteria

Exclusion criteria included non-English publications, articles without full text, and studies not focused on smoking reduction.

## Review

Translating behavioural theory into practice: a targeted smoking cessation intervention

The target population of smokers with current chronic respiratory conditions was chosen. There is hope in identifying further barriers that can be identified to highlight where further research is potentially needed to guide public health policy. In a systematic review looking at behaviour change strategies in interventions targeting sedentary behaviour, behaviour change strategies were identified for use in developing future interventions targeting a sedentary lifestyle [22,23].

Research has hypothesised that, as a general rule, for those in an “at-risk population,” 40% of individuals are in the pre-contemplation stage, 40% are in the contemplation stage, and 20% are in the preparation stage [24]. The intervention, therefore, would have the greatest impact targeting one of the earlier stages of the model. As part of the intervention, one effective tool to incorporate is motivational interviewing. It can be defined as “a directive, client-centred counselling style for eliciting behaviour change by helping clients to explore and resolve ambivalence” [25]. It is an effective collaborative approach in supporting patients in self-regulating their behaviours. The combination of motivational interviewing with a Transtheoretical Model-based approach can produce an effective Intervention.

One study looking at combining the two when managing depression as a risk factor in hospitalised patients with coronary heart disease concluded positive effects [26]. Another study, using the same combined approach towards adolescent smoking cessation, found both positive short-term benefits and positive progression through each stage [27]. This paper proposes community “support hubs” offering group sessions and brief motivational interviewing, accessible by referral or self-referral.

The difficulty comes in developing the system to create a persuasive enough message. Similarly, to self-efficacy, decisional balance is a concept based on the model, which is the person’s belief that they will be able to perform a new behaviour, which will substitute the poor health behaviour [5], in this case, smoking. As individuals progress through each stage of the model, their perceived benefits of the behaviour change will start to further outweigh the risks. O’Keefe, however, notes on the research on decisional balance that it had examined the perceived “importance” of benefits and negatives across each stage, rather than the “number” of perceived benefits and negatives. Further, he argued that decisional balance may have been related to various stages of the model, but that does not necessarily mean it was the cause of progressing through each stage [26].

Despite this, O’Keefe noted that a combination of self-efficacy and decisional balance is important to generate persuasive messages based on the respective stage [26]. Self-efficacy questions could include “quitting smoking is easy” and ranking how much an individual agrees. The gathered data could help create positive reinforcing messages, which hopefully increase individuals’ self-efficacy so they can start to plan for and actively change their behaviour (preparation/action). This is important, as higher self-efficacy predicts more proactive engagement and more positive affect during change [27-29].

The added benefits of designing the intervention based on the model are that the idea of relapse would be less detrimental to an individual in the later stages of action and/or maintenance. One further critique of the model notes that the theory does not address the time frame between an individual having an “intent” and an individual’s “behavioural action” [13]. Regardless, the intervention described for smoking cessation assists in generating positive reinforcement to encourage individuals who reach that behavioural action stage, with the least amount of risk and impact from individuals relapsing back into smoking. Alongside this, one can reinforce the negative impact of relapsing on individuals by preparing them for the genuine possibility of relapse at later stages, but with preparation, the impact on the individual could be less so with prior knowledge. Overall, the aim of this intervention is to enable these patients, educate them on their condition and general risks, and utilise relevant theoretical frameworks to produce a greater number of individuals with intent and increase the perception of the benefits of intent in stopping smoking.

The role of social support and community-based delivery in behaviour change

Group-Based Interventions: Mechanisms and Efficacy

Social support is a robust predictor of successful behaviour change, particularly in the realm of addiction control. Group-based cessation programs leverage peer modelling, social norms, and mutual accountability to reduce relapse and improve adherence [30,31]. These interventions offer normalisation of relapse, reinforcement of social norms favouring recovery, and opportunities to model desired behaviours. The group model presents an economically viable strategy for scaling intervention delivery. Compared to one-to-one clinical care, group work allows facilitators to reach more individuals while optimising constrained health system resources, a key priority in overstretched systems [32].

Community Delivery: Accessibility and Equity

The shift towards community-based service delivery aligns with the NHS Long Term Plan and Integrated Care Board (ICB) strategies, emphasising reducing reliance on hospital-based, high-cost settings [33,34]. Locally accessible hubs for behaviour change support enable earlier intervention, relieve pressure on acute services, and improve service accessibility for underserved populations.

Neighbourhood-based delivery models help dismantle concrete barriers to care, such as transportation challenges, financial constraints, and physical disability. Offering services in community centres instead of hospitals can reduce the impacts of structural discrimination and health disparities [35,36]. Individuals with mental illness, mobility limitations, or caregiving responsibilities often face difficulties accessing remote hospital-based services. Community-based care addresses these inequities directly.

Integrated and Function-Informed Support

Community support services must be embedded within broader physical and mental health systems to ensure integrated, function-informed care. Problematic behaviours like smoking often serve adaptive psychological or physiological functions, such as appetite suppression among individuals with body image concerns. Without addressing these underlying determinants, cessation efforts are likely to be incomplete. Seamless referral routes to mental health services, weight management programs, and social care are essential to long-term success [37,38]. In this way, cessation hubs can function as gateways into broader therapeutic ecosystems, where interventions are tailored to the individual’s specific behaviours and life context.

Theoretical Underpinnings and Behavioural Models

The availability and visibility of community-based services also contribute to behaviour change by reducing psychological inertia. According to the Diffusion of Innovation Theory and Transtheoretical Model, the presence of accessible services in one’s immediate environment normalises the idea of change. It can prompt earlier stage movement in individuals not yet ready for action [39]. These services act as “cues to action,” reducing perceived barriers and increasing self-efficacy.

Case Study: The Salford Community Cessation Hub

A practical illustration of this model is the Salford Community Cessation Hub in Greater Manchester. Established as part of an Integrated Care Partnership initiative, this hub offers smoking cessation support in local libraries and community centres, co-located with weight management and mental health services. By removing the need for hospital attendance, the program increased engagement rates by 34% among individuals from socioeconomically deprived backgrounds. Service uptake doubled among individuals with mobility issues and those with primary caregiving responsibilities. The program’s group-based model allowed participants to share experiences and develop peer support networks linked to improved quit rates at six- and 12-month follow-up [40].

The Salford example underscores the potential for decentralised, multi-functional community hubs to not only increase accessibility and uptake but also create environments conducive to sustained behaviour change through social connectedness and integrated care pathways.

Socially Embedded Behaviour Change

Integrating group-based smoking cessation programs into community-based hubs transforms cessation from an isolated clinical event into a socially supported, cohesive process. This approach enhances reach, equity, and sustainability while reducing health inequalities. Scaling up such integrated, community-based models may hold the key to sustainable population-level behaviour change and the real-world application of behavioural science principles [34-41].

## Conclusions

From its inception to recent applications, TTM has demonstrated potential value in smoking cessation. Despite mixed evidence for long-term outcomes, TTM remains a useful conceptual framework when paired with motivational interviewing and community delivery. The framework has appealed to many researchers in various fields, including behavioural medicine, psychology, and public health. Throughout its time of use, the model has gathered widespread criticism. One, as mentioned, is its lack of evidence-based success. It has been suggested that more studies using the model should be conducted. Increasing the number of studies can increase our understanding of this model and its strengths and limitations.

Despite its limitations, the flexibility of this model makes it an effective tool for treating addictions, such as smoking. It can be paired with other cognitive theories to psychologically support and encourage these individuals. It is also important to consider how we measure change. Sometimes what might be considered a “small” change according to research data, e.g., moving to the preparation stage, may have a huge impact on an individual and be the beginning of a bigger lifestyle change.
